# Heat Treatment Promotes Ubiquitin-Mediated Proteolysis of SARS-CoV-2 RNA Polymerase and Decreases Viral Load

**DOI:** 10.34133/2022/9802969

**Published:** 2022-02-23

**Authors:** Yasen Maimaitiyiming, Tao Yang, Qian Qian Wang, Yan Feng, Zhi Chen, Mikael Björklund, Fudi Wang, Chonggao Hu, Chih-Hung Hsu, Hua Naranmandura

**Affiliations:** ^1^Department of Public Health and Department of Hematology of First Affiliated Hospital, Zhejiang University School of Medicine, Hangzhou 310058, China; ^2^Zhejiang University Cancer Center, Hangzhou 310058, China; ^3^Department of Neurobiology and Department of Neurology of the First Affiliated Hospital, Zhejiang University School of Medicine, NHC and CAMS Key Laboratory of Medical Neurobiology, School of Brain Science and Brain Medicine, Zhejiang University, Hangzhou 310058, China; ^4^Zhejiang Provincial Center for Disease Control and Prevention, Hangzhou 310051, China; ^5^State Key Laboratory for Diagnosis and Treatment of Infectious Diseases, Collaborative Innovation Center for Diagnosis and Treatment of Infectious Disease, The First Affiliated Hospital, Zhejiang University School of Medicine, Hangzhou, China; ^6^Zhejiang University-University of Edinburgh (ZJU-UoE) Institute, Haining 314499, Zhejiang, China; ^7^The First Affiliated Hospital, Institute of Translational Medicine, School of Public Health, Zhejiang University School of Medicine, Hangzhou 310058, China; ^8^Hengyang Medical School, University of South China, Hengyang 421001, China; ^9^Women's Hospital, Institute of Genetics, and Department of Environmental Medicine, Zhejiang University School of Medicine, Hangzhou 310006, China

## Abstract

Despite extensive efforts, COVID-19 pandemic caused by the SARS-CoV-2 virus is still at large. Vaccination is an effective approach to curb virus spread, but several variants (e.g., delta, delta plus, omicron, and IHU) appear to weaken or possibly escape immune protection. Thus, novel and quickly scalable approaches to restrain SARS-CoV-2 are urgently needed. Multiple evidences showed thermal sensitivity of SARS-CoV-2 and negative correlation between environmental temperature and COVID-19 transmission with unknown mechanism. Here, we reveal a potential mechanism by which mild heat treatment destabilizes the wild-type RNA-dependent RNA polymerase (also known as nonstructural protein 12 (NSP12)) of SARS-CoV-2 as well as the P323L mutant commonly found in SARS-CoV-2 variants, including omicron and IHU. Mechanistically, heat treatment promotes E3 ubiquitin ligase ZNF598-dependent NSP12 ubiquitination leading to proteasomal degradation and significantly decreases SARS-CoV-2 RNA copy number and viral titer. A mild daily heat treatment maintains low levels of both wild-type and P323L mutant of NSP12, suggesting clinical potential. Collectively, this novel mechanism, heat-induced NSP12 degradation, suggests a prospective heat-based intervention against SARS-CoV-2.

## 1. Main Text

Severe acute respiratory syndrome coronavirus 2 (SARS-CoV-2), the culprit of the coronavirus disease 2019 (COVID-19) pandemic, is highly contagious and transmissible among humans [1]. The virus invades cells through interaction of its spike protein with the cell membrane protein angiotensin-converting enzyme 2 (ACE2) [1]. Although prophylactic/therapeutic vaccines were rapidly developed and widely applied to curb the virus spread, several SARS-CoV-2 variants (e.g., delta, delta plus, omicron (B.1.1.529), and IHU) have been suggested or reported to lead to partial or even complete escape from immune protection provided by vaccination [1–3]. These variants typically carry multiple mutations, especially in the receptor binding domain (RBD) of the spike protein, requiring constant updates for vaccine design [1–3]. In addition, vaccination rates in lower-income areas remain low [4]. Thus, other efficient and accessible strategies are urgently needed.

Fever is a highly conserved defense mechanism of humans and other vertebrates against various infections. Interestingly, bats are rarely affected by the SARS-CoV-2 with mechanisms not yet fully understood [5]. It remains possible that the bat body temperature, which is elevated up to 40°C during flight, might mimic recurrent fever [6]. In addition, accumulating evidence has indicated that increasing environmental temperatures restrain SARS-CoV-2 transmission and decrease the incidence of COVID-19, suggesting temperature sensitivity of SARS-CoV-2 [7–9]. Notably, several studies including ours have revealed remarkable effects of hyperthermia (elevating body temperature beyond normal) or fever in selectively affecting the properties (e.g., stability, posttranslational modification, and ability to interact with other molecules) of oncogenic proteins [10–12]. Taken together, these findings imply that heat treatment might inhibit SARS-CoV-2 virulence through targeting key viral proteins, which merits particular investigation.

**Figure 1:**
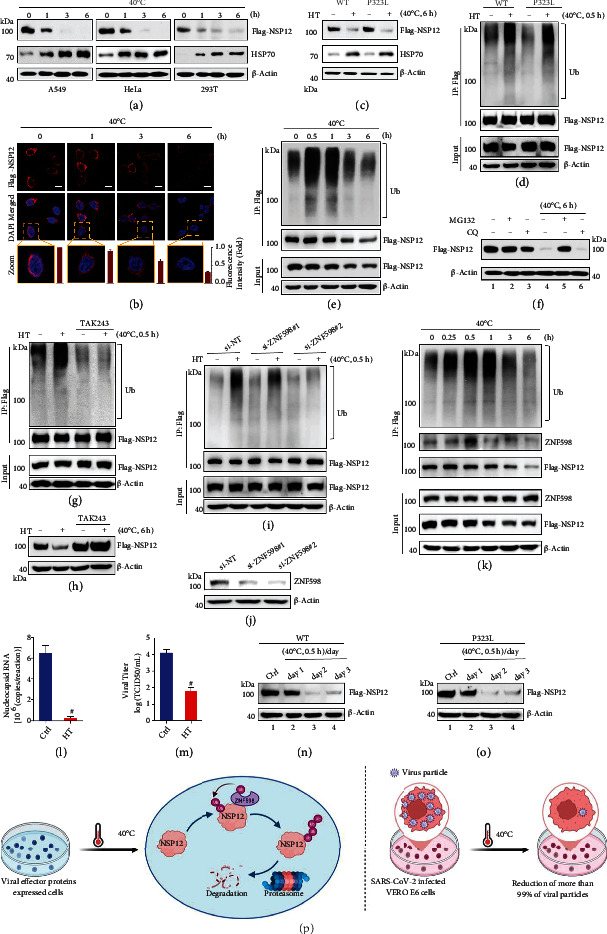
Heat treatment promotes ubiquitin-dependent proteolysis of SARS-CoV-2 RNA polymerase. (a) Time-course study of heat treatment-mediated destabilization of SARS-CoV-2 RNA polymerase (NSP12) in A549, HeLa, and 293T cells. (b) Confocal microscopy analysis of NSP12 protein levels upon heat treatment in NSP12 stably expressing 293T cells. Scale bar is 10 *μ*m. The relative fluorescence intensity of each cell was determined by ImageJ and normalized to control; data shown is mean ± standard deviation (SD) (*n* = 15). (c) Western blot analysis of control and heat-treated (HT) wild-type (WT) and P323L mutant of NSP12 in 293T cells. (d) Heat-induced ubiquitination of WT and P323L mutant of NSP12. NSP12-expressing 293T cells were heat treated as indicated and subjected to immunoprecipitation (IP) analysis. (e) Time-dependent ubiquitination of stably expressed NSP12 in 293T cells, as determined by IP analysis with anti-Flag antibody. (f) Determination of NSP12 degradation pathway by heat treatment. Flag-NSP12 stably expressing 293T cells were pretreated with 10 *μ*g/ml cycloheximide (CHX) with or without 10 *μ*M MG132/20 *μ*M chloroquine (CQ) for 1 h and subjected to heat treatment as indicated. NSP12 protein levels were determined by western blot. Inhibition of NSP12 ubiquitination and degradation by TAK243. 293T cells stably expressing Flag-NSP12 were pretreated with 1 *μ*M TAK243 for 1 h. Ubiquitination of NSP12 was determined by immunoprecipitation (g); NSP12 protein levels were determined by western blot (h). Inhibition of heat induced NSP12 ubiquitination by depletion of ZNF598, as analyzed by IP assay (i); knockdown efficiency of ZNF598 was determined by western blot (j). (k) Changes of NSP12 interaction with ZNF598 upon heat treatment. 293T cells stably expressing Flag-NSP12 were heat treated and subjected to IP analysis. Effect of heat treatment on SARS-CoV-2 viral RNA load and virus titer. SARS-CoV-2-infected VERO E6 cells were heat treated at 40°C for 24 h and subjected to (l) RT-qPCR analysis as well as (m) viral titer analysis. Statistical analysis was carried out using unpaired *t*-test, # represents *p* < 0.001. Compared to the control group, averagely 25.9-fold downregulation of nucleocapsid gene and 218.8-fold reduction of viral titer were observed in the HT group. Data shown is mean ± SD (*n* = 3). (n, o) Effect of daily heat treatment on WT and P323L mutant NSP12 stably expressed in 293T cells. (p) A schematic representation of the mechanism by which mild heat treatment destabilizes the RNA polymerase of SARS-CoV-2 and decreases viral titer.

In this work, we sought to clarify whether viral proteins in SARS-CoV-2-infected cells are vulnerable to mildly elevated temperatures. SARS-CoV-2 nonstructural proteins (NSPs) are the main effectors produced immediately following virus infection as cleavage products of the replicase polyproteins, which are encoded directly from open reading frame 1a and 1ab of the viral genome [13]. NSP12 is also known as RNA-dependent RNA polymerase (RdRp), which forms complex with NSP7 and NSP8 to regulate viral RNA replication and transcription [13, 14]. Nucleocapsid (N) protein is a SARS-CoV-2 structural protein, which is also implicated in the viral RNA replication [15]. Among these effectors, only NSP12 displayed a clear thermal instability via a temperature- and time-dependent manner in multiple human cell lines (Figure 1(a); Figures S1 and S2). Immunofluorescence analysis revealed remarkable reduction of NSP12 levels by heat treatment as well (Figure 1(b)). Notably, NSP12 transcript levels and cell viability were not affected upon these mildly elevated temperatures (Figure S3), suggesting that heat-mediated downregulation of NSP12 resulted from decreased protein stability. As a control, heat treatment increased HSP70 protein or/and mRNA expression (Figure 1(a); Figures S1–S3). In comparison to the spike protein, NSP12 is much less mutation-prone, although one mutation, P323L, has been concurrently identified on NSP12 in several SARS-CoV-2 variants including delta, delta plus, omicron (B.1.1.529), and IHU [1–3]. Notably, we found that P323L mutant and wild-type (WT) NSP12 display similar heat sensitivity (Figure 1(c)), suggesting that heat treatment could be a potential intervention against SARS-CoV-2 variants regardless of their RNA polymerase mutation status.

Next, we investigated the mechanisms involved in heat-mediated downregulation of NSP12. Both WT and P323L mutants of NSP12 were robustly ubiquitinated upon heat treatment within 0.5 h, implying ubiquitin-dependent degradation of NSP12 by the mild heat stress (Figure 1(d)). Gradual reduction of heat-mediated ubiquitination and total NSP12 supported this notion (Figure 1(e)). The proteasome inhibitor MG132 but not the lysosome inhibitor chloroquine (CQ) suppressed heat-induced NSP12 downregulation (Figure 1(f)), further suggesting that heat stress promotes NSP12 degradation through the ubiquitin-proteasome pathway. Additionally, pretreatment with TAK243, a small molecule inhibitor of ubiquitin-activating enzyme, completely inhibited NSP12 ubiquitination (Figure 1(g)) and subsequent degradation (Figure 1(h)) upon heat treatment, supporting the hypothesis that induction of NSP12 ubiquitination is the crucial event for heat-mediated NSP12 degradation.

We next investigated which E3 ubiquitin ligase is involved in heat-mediated degradation of NSP12. Mass spectrometry analysis identified four ubiquitin E3 ligases, ZNF598, STUB1, UBR5, and UHRF1, among 864 potential NSP12 interacting proteins (Table S1; Figure S4a). Of these, knockdown of ZNF598 largely suppressed heat-induced NSP12 ubiquitination (Figure S4b; Figures 1(i) and 1(j)), and interaction of ZNF598 with NSP12 was rapidly increased and subsequently reduced upon heat treatment (Figure 1(k)). These data therefore suggest that ZNF598 is the potential E3 ligase for heat-stimulated ubiquitination of NSP12.

We next validated the effect of heat treatment on SARS-CoV-2 virulence. SARS-CoV-2-infected VERO E6 cells were incubated at 40°C for 24 h and then subjected to qPCR as well as viral titer analysis. Compared to the control group, a more than 20-fold reduction of nucleocapsid gene level was observed in the heat treatment group (Figure 1(l)). Consistently, viral titer in the heat treatment group was also significantly decreased (reduction more than 99.5%) (Figure 1(m)). These results suggest that heat treatment-induced degradation of NSP12 leads to reduction of viral RNA load and downregulation of viral titer. Notably, daily mild heat treatment (40°C, 0.5 h/day) is sufficient to maintain low levels of both WT and P323L mutant of NSP12 (Figures 1(n) and 1(o), compare lanes 3, 4 to 1), suggesting clinical potential of heat treatment against both WT and P323L mutation harboring SARS-CoV-2 variants. In addition, it is demonstrated that mild heat treatment upregulates the immune function [16, 17], which might contribute to inhibition of SARS-CoV-2 fitness as well. Comorbidities such as chronic diseases and acute organ injuries are strongly correlated with disease severity and mortality among COVID-19 patients [18]. Since SARS-CoV-2 mainly infects the lower respiratory tract [1], local daily heat treatment of the airways and lung by available approaches such as radiofrequency hyperthermia [19] may have feasibility in restraining SARS-CoV-2 virulence and controlling severity of the disease.

Heat treatment in the form of sauna or hot bath is a facile, widely accessible, and inexpensive approach practiced widely for therapeutic and recreational purposes, making it a quickly scalable measure for emerging new variants. In fact, mild heat treatment has already been applied in the management of other diseases like cancer, wound, and microbial infection [20, 21]. Our recent work also demonstrated heat treatment as an efficient approach to destabilize thermal-sensitive oncogenic proteins in acute promyelocytic leukemia patients with clinical benefits [12]. In this work, we have identified heat vulnerability of SARS-CoV-2 RNA-dependent RNA polymerase (NSP12) and explained its molecular mechanism. Our results potentially provide a mechanistic insight into recent observations that COVID-19 patients with higher body temperature at the initial presentation show lower mortality rate [22, 23], that bats are rarely affected by SARS-CoV-2 infection [5], and that increasing environmental temperature restrains COVID-19 transmission rate [7–9].

Several potential challenges exist for clinical application of mild heat treatment. First, the number of hyperthermia equipment is limited in the hospitals. Second, proper heating instrument should be selected to achieve efficient and specific heating of the infected region. Third, patients might present unexpected adverse events including fatigue, dizziness, and vomiting, and in such circumstances, the treatment should be ceased. Thus, heat-based treatment of the patients should be conducted under professional supervision. Although our study lacks adequate evidence obtained from *in vivo* animal model showing the inhibitory effect of mild heat treatment on the SARS-CoV-2 virulence, we do provide clear evidence that mild daily treatment is sufficient to maintain low levels of both WT and P323L mutant of NSP12. Hence, fever-range and clinically relevant hyperthermia-based approaches could be rapidly developed for currently prevalent and emerging SARS-CoV-2 variants harboring P323L mutation, including delta, delta plus, omicron, and IHU.

## Data Availability

All data is available in the main text or the supplementary materials.

## Supplementary Materials

Supplementary Materials Materials and Methods Figure S1: heat treatment destabilizes NSP12 in multiple cell lines. Figure S2: NSP7, NSP8, and N proteins show resistance to heat treatment. Figure S3: heat treatment destabilizes NSP12 without compromising cell viability and altering NSP12 transcript levels. Figure S4: mass spectrometry identification and validation of ubiquitin E3 ligase involved in heat-mediated NSP12 degradation. Table S1: complete list of proteins interacting with NSP12 determined by immunoprecipitation coupled with mass spectrometry.
