# Supplementary material for: Heat Treatment Promotes Ubiquitin-Mediated Proteolysis of SARS-CoV-2 RNA Polymerase and Decreases Viral Load
Source: Research (Wash D C). 2022 Feb 23;2022:9802969. doi: 10.34133/2022/9802969 (PMC8918953; doi:10.34133/2022/9802969)

Research
A SCIENCE PARTNER JOURNAL
SPJ
SCIENCE PARTNER JOURNALS

Supplementary Materials for

# Heat Treatment Promotes Ubiquitin-Mediated Proteolysis of SARS-CoV-2 RNA Polymerase and Decreases Viral Load

Short Title: Heat Treatment Destabilizes SARS-CoV-2 RNA Polymerase

Yasen Maimaitiyiming[1,2,3]†, Tao Yang[1,2]†, Qian Qian Wang[1,2]†, Yan Feng[4], Zhi Chen[5], Mikael Björklund[6], Fudi Wang[7,8], Chonggao Hu[4*], Chih-Hung Hsu[9*], Hua Naranmandura[1,2*]

[1]Department of Public Health, and Department of Hematology of First Affiliated Hospital, Zhejiang University School of Medicine, Hangzhou 310058, China.
[2]Zhejiang University Cancer Center, Hangzhou 310058, China.
[3]Department of Neurobiology and Department of Neurology of the First Affiliated Hospital, Zhejiang University School of Medicine, NHC and CAMS Key Laboratory of Medical Neurobiology, School of Brain Science and Brain Medicine, Zhejiang University, Hangzhou 310058, China.
[4]Zhejiang Provincial Center for Disease Control and Prevention, Hangzhou 310051, China.
[5]State Key Laboratory for Diagnosis and Treatment of Infectious Diseases, Collaborative Innovation Center for Diagnosis and Treatment of Infectious Disease, The First Affiliated Hospital, Zhejiang University School of Medicine, Hangzhou, China.
[6]Zhejiang University-University of Edinburgh (ZJU-UoE) Institute, Haining 314499, Zhejiang, China.
[7]The First Affiliated Hospital, Institute of Translational Medicine, School of Public Health, Zhejiang University School of Medicine, Hangzhou 310058, China.
[8]Hengyang Medical School, University of South China, Hengyang 421001, China.
[9]Women's Hospital, Institute of Genetics, and Department of Environmental Medicine, Zhejiang University School of Medicine, Hangzhou 310006, China.
†These authors contributed equally to this work.

* Correspondence to:
Dr. Chonggao Hu, Zhejiang Provincial Center for Disease Control and Prevention, Hangzhou 310051, China, Email: chghu@cdc.zj.cn.

Dr. Chih-Hung Hsu, Women's Hospital, and Institute of Genetics, and Department of Environmental Medicine, Zhejiang University School of Medicine, Hangzhou, Zhejiang, 310058, China, E-mail: ch_hsu@zju.edu.cn.

Dr. Hua Naranmandura, Department of Public Health, and Department of Hematology of First Affiliated Hospital, Zhejiang University School of Medicine, Hangzhou, Zhejiang, 310058, China, E-mail: narenman@zju.edu.cn.

## 1. Materials and Methods

**Cell lines and primary cultures.** 293T, A549 and HeLa cell lines were purchased from Cell Bank of Chinese Academy of Sciences. Following receipt, cells were grown and frozen as a seed stock as they were available. Cell lines were authenticated using DNA fingerprinting (variable number of tandem repeats), confirmed that no cross-contamination occurred during this study, and all cell lines were tested for mycoplasma contamination once in a year. Experiments regarding SARS-CoV-2 infection of VERO E6 cells were conducted at Zhejiang Provincial Center for Disease Control and Prevention. Cells were cultured and maintained in DMEM (Gibco, 12800-082) medium supplemented with 10% fetal bovine serum (Gibco, 10270-106), 100 U/mL penicillin and 100 μg/mL streptomycin, and kept at 37°C in 5% $CO_2$ atmosphere.

**Reagents and antibodies.** MG132 (S2619), chloroquine (S4157) and TAK243 (S8341) were purchased from Selleck. Flag antibody (F1804) was purchased from Sigma. RFP (67378) antibody was obtained from ProteinTech group (Wuhan, China). Ubiquitin (A15546), HSP70 (AF1156), GFP (AG281), and HRP labeled anti-mouse (A0216) as well as anti-rabbit (A0208) secondary antibodies were purchased from Shanghai Beyotime biotechnology. ZNF598 and β-Actin (AC026) antibodies were purchased from ABclonal (Wuhan, China). Dylight 549 anti-mouse IgG (A23310) antibody was purchased from Abbkine (Wuhan, China).

**Trypan blue cell viability assay.** Cells were subjected to indicated treatments before the assay. Then, cells were washed and trypsinized to obtain single cell suspension. Cells were centrifuged, washed twice and resuspended in PBS. Next, 0.4% trypan blue in PBS solution was mixed with the cell suspension in 1:9 ratio (0.04% trypan blue in the mixture), incubated for 1 min, and observed under a light microscope. Images were taken within 3 min and cell viability was calculated by comparing the number of non-stained cells to the number of all visible cells (stained plus non-stained).

**RNA Extraction and qPCR Analysis.** Total RNA was extracted using TRIzol reagent (Life Technologies) according to the manufacturer's instructions. 1 μg of total RNA from each group of cell sample was used as template for the reverse transcription, which was carried out using a PrimeScript RT reagent kit (Takara, RR037A). The resulting cDNAs were used as templates for quantitative PCR (qPCR) or sequencing analysis. The relative expression levels of specific mRNAs were measured by real-time qPCR using the SYBR green qPCR kit (Takara, DRR820A) and analyzed on a Bio-Rad CFX-96 detection system. Gene-specific primer sequences (5'-3') were NSP12 (Forward: CCGACTTGGTCTATGCCCTC, Reverse: GCTCGCCGAGATTAGCGTAT), Nucleocapsid (Forward: GGGGAACTTCTCCTGCTAGAAT, Reverse: CAGACATTTTGCTCTCAAGCTG), ZNF598 (Forward: CCGGGAGAAGCACTTTCTGT, Reverse: TAGTCTTCGCCACCAACGAC), UBR5 (Forward: GAGACTCTGAGCTGTTGCGT, Reverse: TTGGCTCTCCTTCCTTGCTG), STUB1 (Forward: ACTCGTGGAAACTGCTGGAG,

Reverse: TCAGATCAACCACCACAGGC), UHRF1 (Forward: CAACCACTACGGACCCATCC, Reverse: CCAGGACTAGGGAGTACGCT), HSP70 (Forward: AGGCCGACAAGAAGAAGGTG, Reverse: CCGCTGATGATGGGGTTACA), GAPDH (Forward: AATCCCATCACCATCTTCCA; Reverse: TGGACTCCACGACGTACTCA).

**Protein expression and gene silencing.** Expression vector of SARS-CoV-2 NSP12 was purchased from Hunan Fenghui Biotechnology. The SARS-CoV-2 NSP7 and NSP8 expression vectors were generously provided by prof. H. Eric Xu, CAS Key Laboratory of Receptor Research, Shanghai Institute of Materia Medica, Chinese Academy of Sciences, Shanghai, China. N protein plasmid was a generous gift from prof. Pei-Hui Wang, Cheeloo College of Medicine, Shandong University. For transient expression, Flag or GFP or RFP tagged PCMV expression vectors were used. For stable expression, Flag-tagged pCDH expression vector was used along with helper vectors (psPAX2 and PMD2.G) to produce lentiviral particles. Infected cells were selected with puromycin to obtain stable clones. Gene silencing was performed by siRNA transfection into cells using lipofectamine 3000 (Invitrogen, L3000-008) according to the manufacturer's instructions. 24~36 h after transfection, cells were treated as indicated and subjected to various analysis. The target sequence of ZNF598 siRNAs were: si-ZNF598 (#1, CAGGACTACTACAGCGACTAT; #2, GACAATGATGAGCTGCTTAAG, si-UBR5 (#1, TTGGAACAGGCTACTATTAAA; #2, GCTGTAGATTTCAACTTAGAT), si-STUB1 (#1, CCCAAGTTCTGCTGTTGGACT; #2, GAAGAG GAAGAAGCGAGACAT), si-UHRF1 (#1, CCGCACCAAGGAATGTACCAT; #2, ATGTGGGATGAGACGGAATTG).

**Protein extraction and western blot analysis.** Cells were washed twice with D-Hank's solution, followed by lysis using 7 M urea in RIPA lysis buffer (50 mM Tris, 150 mM NaCl, 1% NP-40, 0.5% sodium deoxycholate, 0.1% SDS, pH 7.5, 0.2 mM PMSF, and a complete mini protease inhibitor tablet) to obtain total cell lysates. Samples were incubated on ice for 30 min with vortexes in 10 min interval and centrifuged for 30 min at 4°C, 13000 rpm to obtain the supernatant for western blot analysis. Protein concentrations were measured using BCA Protein Quantification Kit (Yeasen Biotech, 20201ES76). 25 μg of each protein sample was resolved by 7.5~12% SDS-PAGE and blotted onto PVDF membranes. The membranes were blocked with non-fat milk and incubated overnight with different primary antibodies at 4°C, followed by incubation with HRP-labeled secondary antibodies for 1 h at room temperature. Then protein bands were visualized by enhanced chemiluminescence (Biological Industries, 20-500-120).

**Immunoprecipitation and proteomics analysis.** Cells were seeded in 10-cm culture dishes and subjected to indicated treatments. Then, cells were scraped down and collected in ice-cold PBS, lysed on ice by sonication in IP buffer (50mM Tris-HCl pH7.5, 10% glycerol, 150 mM NaCl, 2 mM EDTA, 0.5% NP-40, 1 mM PMSF plus protease inhibitors). Immunoprecipitation experiments were performed using Protein A/G PLUS-Agarose Immunoprecipitation Reagent (CST, sc-2003) according to the manufacturer's

instructions. The immunoprecipitated proteins were subjected to western blot analysis or proteomics analysis. Proteomics (Mass-Spectrometry) experiments were performed by Aksomics, Shanghai.

**Immunofluorescence microscopy.** Cells were grown in culture plates or wells containing glass disks and transferred onto glass slides following indicated treatments. Slides were washed twice with PBS, fixed in 4% paraformaldehyde and permeabilized with 0.1% Triton X-100. Slides were blocked with 2% BSA in PBS, followed by incubation with primary antibodies overnight at 4°C. Next day, slides were washed thrice with PBS and incubated with fluorescent-labeled secondary antibodies (Table S7) at room temperature for 4 h and washed thrice with PBS. Slides were mounted using DAPI Fluoromount-G® (SouthernBiotech, 0100-20) and stored in dark at 4°C. The fluorescent signals were visualized under a Zeiss (Göttingen, Germany) 510 confocal microscope.

**Statistical Analysis.** Each experiment was performed at least three times. Statistical analysis was carried out using unpaired t-test (Sigmaplot, Systat Software Inc), and a probability value of less than 0.05 (*$p<0.05$) was accepted as a significant difference, ** represents $p<0.01$, # represents $p<0.001$.

## 2. Supplementary Figure Legends and Figures

**Supplementary Figure 1. Heat treatment destabilizes NSP12 in multiple cell lines.** (**a**-**c**), Flag-NSP12 Transfected cells were subjected to heat treatment as indicated, changes in protein levels were determined by western blot.

**Supplementary Figure 2. NSP7, NSP8 and N proteins shows resistance to heat treatment.** (**a**), Schematic illustration of SARS-COV-2 RNA polymerase complex, which is composed of NSP7, NSP8 and NSP12. (**b** and **c**), GFP-NSP7 and RFP-NSP8 transfected 293T cells were subjected to heat treatment as indicated, changes in protein levels were determined by western blot. (**d**), Schematic representation of SARS-CoV-2 structural proteins. (**e**), Flag-N protein transfected 293T cells were subjected to heat treatment as indicated, changes in protein levels were determined by western blot. Figures A and D were created on BioRender website (BioRender.com).

**Supplementary Figure 3. Heat treatment destabilizes NSP12 without compromising cell viability and altering NSP12 transcript levels.** (**a**-**d**), Flag-NSP12 stably expressing 293T cells were subjected to heat treatment (HT) as indicated, changes in protein levels were determined by western blot; cell viability was determined by trypan blue assay; relative expression of NSP12 and HSP70 transcripts were detected by RT-qPCR. Data shown is mean ± SD, n=3.

**Supplementary Figure 4. Mass spectrometry identification and validation of ubiquitin E3 ligase involved in heat-mediated NSP12 degradation.** (**a**), List of the E3 ubiquitin ligases among 864 potential NSP12 interacting partners identified by mass spectrometry. (**b**), Identification and validation of ubiquitin E3 ligase involved in heat-mediated NSP12 degradation. Two siRNAs to each ubiquitin E3 ligase gene were mixed in equal amount and transfected into Flag-NSP12 stably expressing 293T cells, then subjected to heat treatment. Changes in NSP12 ubiquitination level were determined by IP assay. Knockdown efficiency of siRNAs was assessed by RT-qPCR. Data shown is mean ± SD, n=3.

# Supplementary Figure 1

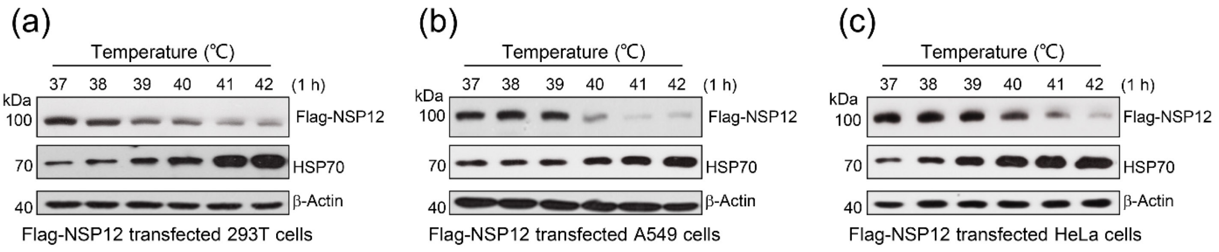


# Supplementary Figure 2

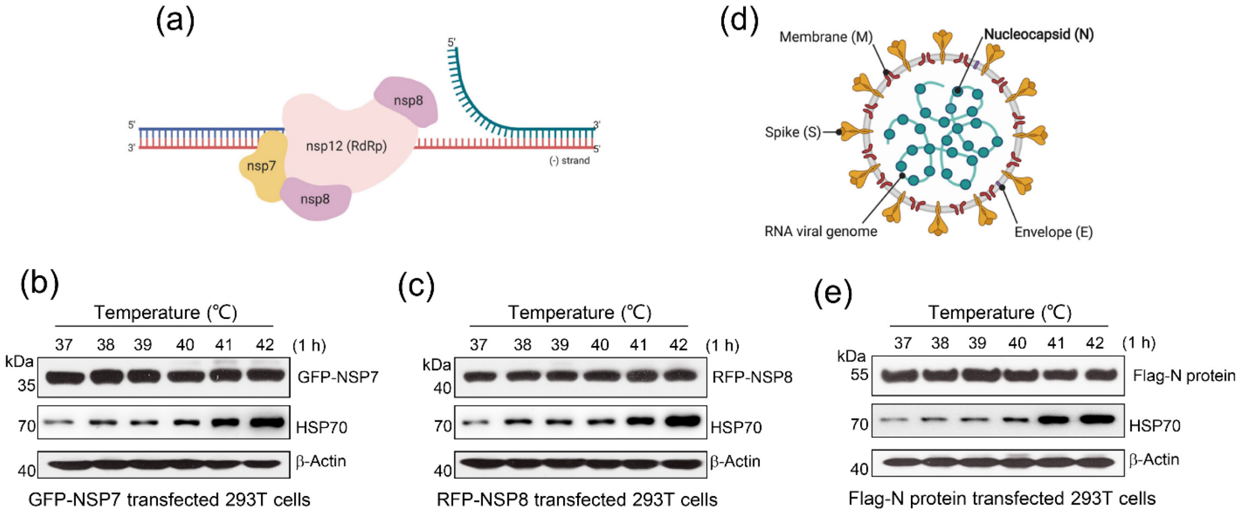


# Supplementary Figure 3

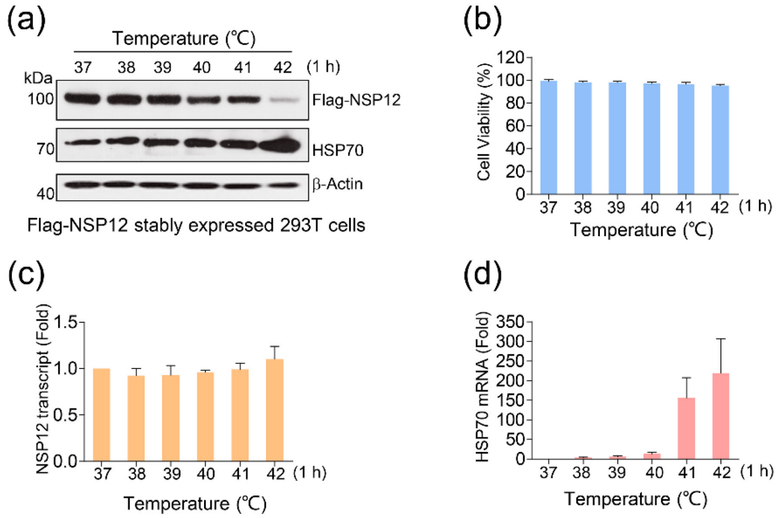

## Supplementary Figure 4

(a)

| Gene Name | Score | Sequence Coverage | Protein Size (AA) |
|---|---|---|---|
| STUB1 | 3.3982 | 12.5 | 303 |
| UBR5 | 3.2929 | 1.1 | 2799 |
| ZNF598 | 2.3291 | 2.8 | 904 |
| UHRF1 | 1.9173 | 3.6 | 806 |

(b)

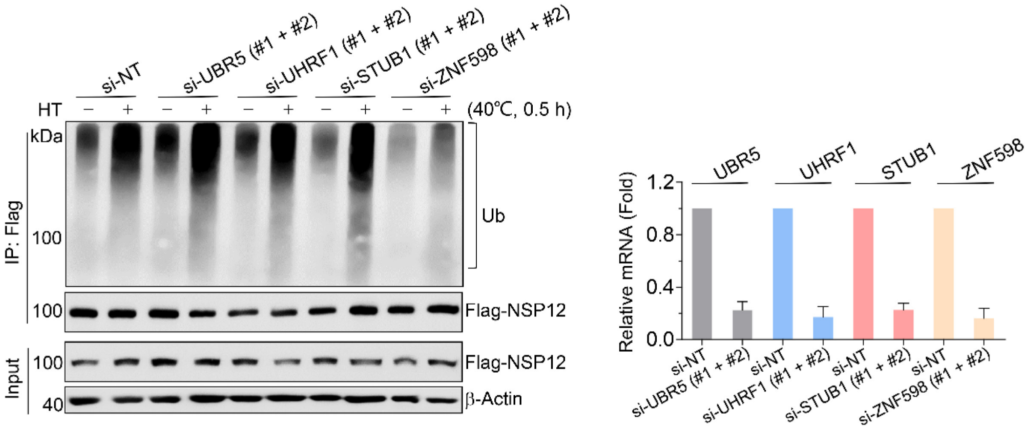

Supplement: Supplementary Materials — Materials and Methods Figure S1: heat treatment destabilizes NSP12 in multiple cell lines. Figure S2: NSP7, NSP8, and N proteins show resistance to heat treatment. Figure S3: heat treatment destabilizes NSP12 without compromising cell viability and altering NSP12 transcript levels. Figure S4: mass spectrometry identification and validation of ubiquitin E3 ligase involved in heat-mediated NSP12 degradation. Table S1: complete list of proteins interacting with NSP12 determined by immunoprecipitation coupled with mass spectrometry. [file 9802969.f1.zip › Supplementary Materials.pdf]
