# Supplementary material for: Heat Treatment Promotes Ubiquitin-Mediated Proteolysis of SARS-CoV-2 RNA Polymerase and Decreases Viral Load
Source: Research (Wash D C). 2022 Feb 23;2022:9802969. doi: 10.34133/2022/9802969 (PMC8918953; doi:10.34133/2022/9802969)
Supplement: Supplementary Materials — Materials and Methods Figure S1: heat treatment destabilizes NSP12 in multiple cell lines. Figure S2: NSP7, NSP8, and N proteins show resistance to heat treatment. Figure S3: heat treatment destabilizes NSP12 without compromising cell viability and altering NSP12 transcript levels. Figure S4: mass spectrometry identification and validation of ubiquitin E3 ligase involved in heat-mediated NSP12 degradation. Table S1: complete list of proteins interacting with NSP12 determined by immunoprecipitation coupled with mass spectrometry. [file 9802969.f1.zip › Supplementary Table 1.pdf]

Supplementary Table 1. Complete List of Proteins Interacting with NSP12 Determined by Immunoprecipitation Coupled with Mass Spectrometry.

Gene Name	Score	Sequence.coverage....	protein_size
ABCF1	8.687	8.9	845
ACAT1	12.676	27.6	427
ACAT2	1.8424	5	397
ACTB	323.31	61.6	375
ADAR	6.3042	8.2	1226
PARP1	323.31	51.4	1014
ADSL	7.3918	16.7	484
AHCY	9.5153	12.3	432
ALDH1B1	9.304	11.8	517
ALDOA	18.647	25.5	418
AMPD2	1.1907	1.6	879
SLC25A5	53.865	35.6	298
SLC25A6	19.658	40.6	298
ANXA2	4.2843	12.1	357
APRT	8.7323	23	180
ARF1	85.605	54.1	181
ARF4	8.1347	50.6	180
ARF6	1.1841	12.6	175
RHOA	259.61	43.5	193
ARL1	1.1606	64.7	181
ARL2	3.6375	16.8	184
ASNS	6.5316	16.9	561
ATP1A1	89.759	21.6	1023
ATP2A2	19.762	14.2	1042
ATP5A1	140.46	37.4	553
ATP5B	47.866	26.5	529
ATP5C1	17.629	24.6	298
ATP5F1	4.5022	17.9	256
ATP5O	40.653	46.5	213
BLVRA	2.4593	8.1	296
BSG	8.0386	35.4	385
BYSL	4.7161	11.4	437
C1QBP	57.801	9.6	282
CAD	22.162	9.6	2225
CALM2	12.351	30.9	149
CALM2	12.351	30.9	149
CALR	8.3603	19.4	417
CANX	22.15	19.1	592
CAPZA1	15.93	25.9	286
CCNT1	1.7845	6.2	726
CCT6A	16.252	20	531
CDC2	14.51	32.3	297
CDC42	3.3434	23.6	191
CENPB	19.878	6.5	599
CFL1	39.172	40.9	166
RCC1	28.994	24.2	452
CHD1	37.265	10.5	1710
CHD4	4.8177	4.6	1912
CKB	12.529	16	381
TPP1	1.3204	14.1	563
CLTC	129.51	17.9	1679
COPA	33.511	17.6	1233
COPB1	10.123	10.1	953
CS	3.2607	7.8	466
CSE1L	12.186	5.7	971
CSNK2B	2.1065	8.5	215
SLC25A10	15.52	33.1	442
SLC25A10	15.52	33.1	406
CSTB	3.0616	24.5	98
CTNND1	14.541	10.6	968
CTPS1	24.391	23.8	591
CTSD	3.9841	15.2	412
CYC1	7.523	11.7	325
DARS	29.331	34.5	501
DCTN1	27.872	9.2	1278
DDB1	35.919	7.8	1140
DDOST	23.173	7.1	456
DDX1	25.335	12.4	740
DDX3X	94.244	36.3	662
DDX5	70.543	34.7	614
DDX6	4.1248	8.9	483
DHX9	44.682	18.1	1270
DHX15	15.712	19.7	795
DHCR7	8.2931	13.3	475
DKC1	19.018	18.3	514
DLD	3.3811	10.1	509
DYNC1H1	32.055	5.8	4646
DNM2	19.03	11	870
DSC1	6.5422	8.1	894

DSG1	15.765	7.3	1049
DSP	43.831	10.8	2871
DUT	2.9258	21.3	252
EEF1A1	261.32	31.8	462
EEF1B2	2.5962	13.8	225
EEF1D	26.958	43.8	647
EEF1G	75.568	37.1	437
EEF2	41.833	31.6	858
EIF2S1	5.4371	13.3	315
EIF2S3	23.4	23.1	472
EIF4A1	323.31	57.6	406
EIF4E	2.8152	10.2	248
EIF4G1	20.811	12.1	1606
EIF5A	6.7961	32.2	184
ELAVL1	6.8553	16.6	326
EMD	46.588	41.7	254
ENO1	237.23	40.3	434
EPB41	3.7315	7.2	864
EPB41L2	16.189	11.9	1005
EPRS	71.587	25.9	1512
ERH	227.36	41.3	104
FBL	54.709	30.5	321
ESD	2.5542	15	282
ETFA	10.409	19.8	333
EWSR1	4.4367	9.4	661
FABP5	1.164	10.9	135
FARSA	132.22	30.1	508
FASN	108.17	17.9	2511
FDPS	14.999	13.9	419
FEN1	47.88	17.4	380
FHL1	13.805	24.5	323
FHL3	1.6955	7.1	280
FLII	9.7929	5.5	1269
FLNA	85.351	20.8	2647
FLNB	16.448	8.6	2633
FUS	33.487	13.1	526
XRCC6	15.733	24.2	609
GALK1	4.9295	15.8	392
GAPDH	292.87	56.7	335
GARS	6.7135	6	739
GART	33.891	13.3	1010
GLA	19.235	22.1	429
GNAS	2.7466	10.6	1037
GNB1	3.4719	13.9	340
GOT2	2.2782	6.7	430
GPX1	5.5227	24.8	203
GRN	17.836	13.8	593
GSTP1	11.571	22.4	210
MSH6	11.366	4.7	1360
GTF2I	49.022	11.9	998
H1F0	12.449	27.8	194
HIST1H1C	98.35	39	213
HIST1H1D	3.3354	34.4	221
HIST1H1T	3.0921	22.7	207
HIST1H2BC	323.31	83.3	126
HIST1H2BC	323.31	83.3	126
HIST1H2BC	323.31	83.3	126
HIST1H2BC	323.31	83.3	126
HIST1H2BC	323.31	83.3	126
HIST1H2BC	323.31	83.3	126
HADHA	34.878	25.9	763
HADHB	5.4038	8.5	474
HCFC1	6.7144	5.6	2035
HDAC1	6.8494	13.5	482
HDAC2	1.9615	12.2	488
HELLS	12.804	11.2	884
HK2	5.9904	2.5	917
HMGB1	5.0434	25.9	215
HMGN1	7.2901	37	100
HMGN2	4.939	32.2	90
P17096	5.9609	23.4	107
HNRNPA1	84.191	56.4	372
HNRNPA2B1	104.67	53	353
HNRNPAB	5.6526	14.3	332
HNRNPC	71.033	46.6	306
HNRNPD	56.088	30.8	355
HNRNPF	92.088	38.3	415
HNRNPH1	323.31	42.2	449
HNRNPH2	40.38	31	449
HNRNPH3	6.2992	12.1	346
HNRNPK	323.31	40.9	464

HNRNPL	56.75	29.7	589
HNRNPU	323.31	32.7	825
HPRT1	5.0514	31.7	218
HSD17B4	7.0524	19	761
DNAJA1	39.785	37	397
HSPA1B	156.2	36.8	641
HSPA5	191.25	41.3	654
HSPA6	1.987	14.2	643
HSPA8	261.68	47.7	646
HSPA9	83.966	42.7	679
HSP90AA1	155.59	36.1	854
HSP90AB1	226.82	37.7	724
HSPD1	165.34	32.5	573
HSPE1	4.3633	35.3	102
IARS	39.892	13.4	1262
IDH1	2.4188	12.1	414
IDH2	16.365	27	452
IDH3A	6.4172	21.3	366
IDH3B	4.5301	7.5	385
IK	4.6095	5.6	557
ILF2	22.729	33.8	390
ILF3	22.014	21.6	898
ILK	3.2282	7.2	452
IMPDH2	24.045	33.8	514
INCENP	4.7572	6.5	918
EIF3E	3.8073	14.2	445
STT3A	6.2519	7.4	705
ITPA	2.6169	9.6	194
JUP	19.285	12.6	745
KIF5B	10.251	10.1	963
KPNB1	51.426	9.7	876
KPNA2	79.393	22.5	529
KRT18	6.638	15.3	430
RPSA	110.2	27	295
LBR	7.8815	23.1	615
LDHA	21.564	26.6	361
LDHB	10.314	22.3	334
LGALS3	2.2212	4.4	250
LIG3	4.3574	3.7	1009
LMNA	10.245	19.6	664
LMNB1	66.47	31.2	586
LPL	16.184	7.6	475
MAP4	10.866	15.8	1152
MARS	19.326	6.4	900
MAT2A	7.5848	18.7	395
MAZ	2.9863	14.9	493
MCM3	29.926	19.1	853
MCM4	55.787	26.9	863
MCM5	7.4493	12.7	734
MCM6	15.802	15	821
MCM7	79.914	40.9	719
MDH1	1.7234	12.9	352
MDH2	4.1068	14.3	338
RAB8A	12.411	24.6	207
MIF	3.319	17.4	115
MKI67	323.31	41.9	3256
MRE11A	1.9215	5.9	708
MTHFD1	70.238	36	935
MYH9	100.37	23	1960
MYH10	69.544	17.3	2007
MYL6	24.414	38.6	151
NACA	8.5153	40.8	925
HNRNPM	231.94	51.5	730
NAP1L1	24.559	28.8	391
NARS	4.8915	2.6	548
NCL	32.534	15.2	710
NDUFA4	2.6871	27.2	81
NDUFA7	5.2492	23.9	113
NDUFA10	1.4668	5.9	355
NDUFB10	5.9908	28.6	172
NDUFS1	9.8592	12.5	741
NDUFS2	15.208	18.6	463
NDUFS3	17.323	36.4	264
44441	2.1492	17	396
RPL10A	14.681	35.5	217
NFATC1	1.0551	3.1	943
NHP2L1	31.201	18.2	128
NME1	6.4561	28.3	177
NOP2	8.3102	8.7	845
NONO	48.983	32.7	471
NPM1	249.24	35.1	294

YBX1	33.089	41.4	324
NSF	4.3322	4.5	744
NUMA1	66.901	12.5	2115
NUP98	3.5747	2.9	1800
OAT	19.948	23.5	439
OXA1L	2.524	6.4	495
PRDX1	51.067	68.8	199
PCBP1	28.935	30.3	356
PCBP2	13.524	27.4	366
PCMT1	18.334	26.4	286
PCNA	253.63	35.2	261
PCOLCE	2.2556	4.9	449
PDHA1	31.134	37	428
PDHB	28.065	32.8	359
PFKL	4.4353	9.2	780
PFN1	1.7009	13.5	140
PGAM2	3.0234	17.8	253
PGM3	4.2696	7.2	570
PHB	6.2459	22.4	272
SLC25A3	79.932	26	362
PIP	4.9706	26.7	146
PKM	323.31	55.9	531
PNN	77.163	24.3	717
POLR2C	1.1407	21.7	275
POLR2H	1.7999	8.8	175
PPIA	6.1816	33.9	165
PPM1B	162.99	40.9	479
PPM1G	4.3567	6.2	546
PPP1CC	21.524	32.2	337
PPP2CA	75.28	26.5	309
PPP2R1A	15.895	10	589
PPP6C	4.2258	11.7	342
PRKDC	233.4	18.2	4128
PRPS1	22.491	40.4	318
HTRA1	56.178	16.9	480
PSMA1	12.964	36.5	269
PSMA2	33.663	25.2	234
PSMA3	16.415	26.6	255
PSMA4	11.12	22.2	261
PSMA5	29.819	29	241
PSMA6	8.0646	26.6	246
PSMA7	31.44	36.7	248
PSMB1	21.403	32.8	241
PSMB2	2.9168	10.4	201
PSMB3	30.019	16.1	205
PSMB4	7.4537	17.8	264
PSMB5	11.095	27.8	263
PSMB6	1.146	9.8	239
PSMC2	16.136	18.9	433
PSMC3	38.808	22.2	439
PSMC4	10.608	19.6	418
PSMC5	16.983	34	406
PSMC6	6.4741	14.9	403
PSMD1	1.706	3.8	953
PSMD2	22.294	16.3	908
PSMD3	11.311	19.5	534
PSMD4	233.15	43.2	377
PSMD8	2.1702	14.9	350
PSMD11	9.1869	24.6	422
PSMD12	3.4274	9.2	456
PSMD13	3.2188	10.9	378
PTBP1	64.194	22.4	557
RAD1	1.301	3.2	282
ABCD3	8.1655	9.6	659
PYCR1	2.4015	13.6	346
ALDH18A1	1.369	2.6	795
QARS	14.204	15.7	775
RAB1A	32.641	25.4	205
RAB2A	8.339	7.2	212
RAB5A	1.6103	23.9	215
RAB5C	8.4612	24.5	249
RAC1	7.2855	32.3	211
RAN	70.664	43.8	216
RANBP1	8.4786	43	278
RANBP2	10.614	7.4	3224
RANGAP1	1.691	4.8	587
RAP1A	2.5815	23.4	184
RARS	5.8258	12	660
RBBP4	39.891	23.4	425
RBBP7	4.9465	18.5	469
RBM4	110.06	34.3	364

RCN2	11.544	12.3	335
UPF1	8.8841	5.6	1129
RFC1	20.396	9.8	1148
RFC2	3.4037	10	354
RFC3	5.3737	22	356
RFC4	4.429	12.1	363
RNH1	21.775	11.3	461
ABCE1	69.256	22.4	599
RPL3	46.943	38.7	403
RPL4	43.357	41.5	427
RPL5	31.737	29	297
RPL6	58.606	34.7	288
RPL7	45.912	26.2	248
RPL7A	44.335	41	266
RPL8	9.9958	23.7	257
RPL9	84.66	44.3	192
RPL10	64.091	42	214
RPL11	80.579	19.7	178
RPL12	263.04	54.5	165
RPL13	50.75	39.8	211
RPL15	9.2406	34.3	204
RPL17	30.842	46.2	184
RPL18	90.507	43.1	188
RPL18A	49.711	39	176
RPL19	8.0289	28	196
RPL21	84.23	39.1	160
RPL22	3.072	51.1	128
RPL23A	13.827	34.2	156
RPL24	20.571	42.1	157
RPL26	6.0368	32.7	145
RPL27	29.509	38.2	136
RPL30	52.36	51.8	115
RPL27A	6.0016	43.5	148
RPL28	6.0403	37.1	169
RPL29	5.5648	9	159
RPL31	22.646	44.4	128
RPL32	11.543	36.1	135
RPL34	2.8633	23.1	117
RPL35A	2.3334	37.2	110
RPL37A	51.457	58.8	92
RPL38	14.495	50	70
RPL39P5	1.4789	19.6	51
RPL36A	3.1819	24.1	142
RPLP0	66.387	45.4	317
RPLP1	3.5523	14	114
RPLP2	7.5835	53	115
RPN1	116.22	45.6	607
RPN2	70.293	18.4	631
RPS2	30.36	36.5	293
P23396	187.87	71.2	259
RPS3A	92.348	47	264
RPS4X	23.347	42.6	263
RPS5	178.12	51	204
RPS6	24.786	22.9	249
RPS7	6.8396	26.3	194
RPS8	64.453	53.4	208
RPS9	6.5445	28.4	194
RPS10	127.26	44.8	165
RPS11	39.721	52.5	158
RPS12	72.272	53.8	132
RPS13	41.955	46.4	151
RPS14	48.918	40	151
RPS15	1.2638	17.6	145
RPS15A	7.6403	50	130
RPS16	32.803	46.6	146
RPS17	323.31	14.4	135
RPS18	27.414	59.9	152
RPS19	28.24	49.7	145
RPS20	19.511	23.5	142
RPS23	8.5981	29.4	143
RPS24	12.269	35.9	289
RPS25	17.276	24	125
RPS27	32.118	40.5	84
RPS27A	188.87	53.8	156
RPS29	3.3181	20.7	67
S100A8	30.896	39.8	93
S100A9	133.65	37.7	114
SAFB	82.151	28.1	917
SARS	25.509	18.1	514
ATXN2	1.273	4.6	1313
SCD	4.4411	10.6	359

SCO1	1.6088	12.2	301
SDHA	23.424	16	664
SEC13	3.0385	12	368
SFPQ	118.5	29.4	707
SRSF1	23.665	25.3	248
SRSF3	16.969	48.4	164
SRSF6	3.8544	13.3	344
SRSF7	10.602	38.7	238
TRA2B	5.286	31.4	288
SHMT2	1.4566	10.7	504
SKP1	8.8007	7.4	163
SLC3A2	3.1698	3.8	631
SLC16A1	32.181	6.6	500
SLC25A1	6.7945	17.7	311
SMARCA1	2.719	11	1070
HLTF	4.3341	4.3	1009
SMARCA4	4.1136	5.2	1647
SUMO2	10.399	31	95
SNRPC	2.058	6.7	159
SNRPD2	5.2457	39	118
SNRPD3	18.788	50	126
SNRPF	8.6781	24.4	86
SNRPN	3.6461	21.9	240
SON	3.9781	2.4	2426
SRM	3.6427	18.2	302
SRP14	39.744	34.6	136
SRP19	3.0309	12.5	144
SSBP1	4.9469	27.7	148
SSR4	5.0301	19.7	173
SSRP1	131.14	28.3	709
SUPT5H	1.4451	2.4	1087
TAF4	6.4136	6.5	1085
TALDO1	4.4362	9.5	337
TARS	6.7007	9.5	756
TCEB1	1.7547	18.5	112
TCOF1	8.7875	7.3	1488
TCP1	52.72	33.3	556
PRDX2	2.1534	24.2	198
TGM3	1.5183	1.8	693
THBS1	6.6196	7.4	1170
TKT	6.4856	10.7	631
P42166	323.31	43.8	694
P42167	62.253	46.5	694
TOP1	64.513	27.2	765
TOP2A	323.31	41.6	1531
TOP2B	323.31	40.5	1621
TP53	11.598	14.6	393
TPI1	30.047	37.8	286
HSP90B1	17.034	16.2	803
CCT3	115.64	46.4	545
DNAJC7	7.166	11	494
TTN	1.0436	0.1	35991
TUBB2A	4.3006	52.1	445
TUFM	221.01	53.3	455
TXN	10.726	49.5	105
UBA52	1.3885	48.4	128
UBTF	108.38	26	764
UGDH	2.4588	9.1	494
UQCRC1	15.463	16.2	480
UQCRC2	98.725	39.7	453
VARS	23.123	15.4	1264
VCP	4.738	6.2	806
VDAC1	2.9992	12.4	283
VDAC2	25.057	36.2	309
VDAC3	11.189	7.1	284
VIM	323.31	69.3	466
VRK1	10.257	14.6	396
XPO1	10.064	10.6	1071
XRCC1	1.4358	3.3	633
XRCC5	16.538	12.4	732
YY1	4.1633	14.7	414
YWHAB	5.5287	18	246
YWHAE	11.055	25.5	255
YWHAG	6.6153	21.5	247
YWHAZ	227.9	29.4	245
SF1	1.3934	1.8	673
P62633-7	14.439	30.6	179
ZNF229	1.428	5.5	825
CSDE1	3.8155	5.2	844
TUBA1A	38.566	61.1	451
DEK	9.6103	12.1	375

AIMP2	39.551	20	320
MLF2	8.589	25.8	248
AAAS	2.9038	6.4	546
FXR1	3.4991	7.9	621
PABPN1	1.3093	11.5	306
COIL	4.3208	4.5	576
RBM10	3.9371	3.1	995
SMC1A	30.641	13.5	1233
NAA10	1.406	4.5	235
HIST1H2AJ	253.73	41.4	128
HIST1H2AC	1.6559	40.8	130
HIST2H2BE	24.34	83.3	126
SLC25A11	94.967	28.7	314
TAGLN2	1.3014	5.9	220
CUL4B	1.6565	1.3	913
SMARCA5	64.329	29.2	1052
IRS4	96.91	25.9	1257
YARS	9.5796	22.9	528
KHSRP	1.3866	4.2	711
RUVBL1	207.78	48	456
EIF3A	95.68	14.5	1382
EIF3B	22.003	20.5	814
EIF3C	29.33	15	913
EIF3D	9.3194	13.6	548
EIF3F	19.93	14.3	357
EIF3G	12.172	19.4	320
EIF3I	28.719	40.9	325
SRSF9	13.456	31.2	221
BANF1	44.426	50.6	89
GMPS	9.4931	18	693
EIF2S2	5.4463	15.6	333
CPNE8	1.3049	1.6	537
FUBP3	21.615	13.1	572
AP3D1	1.7931	3.7	1215
H1FX	4.8545	12.7	213
BAZ1B	127.15	28.7	1483
UBE2M	2.8208	15.8	183
RPL14	27.037	36.3	215
DNAJA3	6.9161	12.4	480
SMC3	30.086	17.7	1217
AIFM1	32.199	26.3	613
RRP9	2.1853	6.3	475
BUB3	3.7685	13.3	328
DDX21	31.874	22.7	783
AURKB	2.0938	8.3	345
MTA2	1.882	3	668
NOLC1	44.379	19.4	709
TBRG4	1.4106	4	631
AIMP1	4.1215	10.3	336
RPL23	80.184	40	140
MAGED1	5.3704	3.5	834
PMPCB	4.1396	8.2	489
NPEPPS	2.2583	6.5	919
TECR	6.4995	12.4	308
BAG2	7.8245	38.9	211
POLR1C	6.7562	12.4	346
SEC22B	1.3907	4.7	215
H2AFY	227.02	46.3	372
RBM39	58.792	21.3	530
PRDX6	6.8154	22.3	224
NUP155	10.614	6.6	1391
MDC1	323.31	32.2	2089
SAFB2	4.1634	15.5	953
CLINT1	3.5792	8.8	643
NUP93	13.608	16.7	819
EMC2	1.3999	8.9	297
PUM1	13.262	11.9	1188
BCLAF1	28.802	16.2	920
EIF4A3	42.863	41.1	411
MATR3	323.31	35.1	847
ZC3H11A	8.7927	11.8	810
DDX46	4.276	4.2	1032
UBAP2L	10.027	5.5	1087
G3BP2	3.9152	7.6	482
RBM8A	8.9231	32.4	174
THRAP3	76.006	25.2	955
NUP153	20.878	9.1	1506
HNRNPDL	3.3904	8.5	420
CHAF1A	5.2259	9.5	956
SMC4	8.5287	5.2	1288
FARSB	3.7455	7.3	589

ABCF2	17.686	22.5	634
ACTR2	6.4577	9.9	399
ACTR1A	37.506	14.8	376
LRPPRC	12.011	6.9	1394
PDIA6	272.92	33.2	492
TRAP1	1.9842	6.1	704
BCAP31	2.1575	14.2	313
AKAP9	1.0389	0.8	3907
G3BP1	32.541	18.7	466
TRIM28	230.01	36.2	835
SLC25A13	49.806	24.9	676
TENM1	2.1011	0.6	2732
ALYREF	98.889	40.1	264
O00148	18.697	21.8	427
PSMD14	1.8695	4.2	310
HNRNPR	21.214	23.2	636
ZMPSTE24	1.0529	2.3	475
**STUB1**	**3.3982**	12.5	**303**
SAP18	18.453	45.3	172
DNAJA2	13.465	27.7	412
TUBA1B	323.31	56.3	451
TUBB4A	12.316	58.8	444
TUBB4B	323.31	65.8	445
GNB2L1	219.66	63.4	317
PRMT5	2.4232	5.1	637
CDIPT	1.2839	4.2	213
TOMM40	6.2343	14.1	361
HMGN4	2.2993	24.4	90
CAP1	1.3024	5.7	475
SYNCRIP	4.7561	13.5	623
DDX17	53.432	33.9	731
IPO7	2.1569	2	1038
NOP56	28.851	26.6	594
PRDX4	2.3028	26.1	271
SLU7	28.715	10.8	586
CCT7	23.008	29.9	543
CCT4	88.991	34.7	539
CCT2	93.465	35.1	535
SMC2	2.0818	3.4	1197
PRPF8	10.215	4.4	2335
AHSA1	5.3617	10.1	338
PAICS	12.368	22.1	432
TBL3	2.6415	1.9	808
MYL12A	31.924	22	172
MYL12A	31.924	22	171
IGF2BP1	4.087	7.6	577
KHDRBS1	6.9808	9.1	443
CCT8	28.439	35.2	548
POLD3	6.7198	6.9	466
YME1L1	6.6373	6.4	773
KIF1A	1.0488	2.9	1103
HSPH1	4.8008	8.6	858
CLPX	7.8331	15.2	633
SRCAP	10.994	3	3230
RUVBL2	54.053	51.4	463
PGRMC1	4.9579	12.3	195
RAB10	2.3672	16.5	200
TRAFD1	2.5974	5.2	582
MAGED2	15.062	9	606
RNPS1	87.84	33.6	305
SUB1	1.0984	8.7	127
PRDX3	3.4617	10.5	256
SF3A3	11.699	10.2	501
HNRNPA0	50.42	37.7	305
CBX1	13.375	26.8	185
SEC61B	3.2104	37.5	96
CKAP4	12.073	15	602
YWHAQ	59.392	18.4	245
TMED10	15.159	22.4	219
IMMT	41.608	27.6	758
KDELR1	1.0714	5	212
RAB35	1.7414	15.7	201
DSTN	3.4994	25.9	165
CDC37	1.9664	8.7	378
ERLIN2	10.209	22.2	339
O75475	134.26	30.8	530
STRAP	8.5311	22	350
BAZ2A	4.0242	2.2	1905
WDR6	2.2638	2.8	1151
SUPT16H	323.31	33.1	1047
RPL35	5.3054	26.8	123

ATXN2L	7.2769	7.1	1097
PHB2	9.8826	19.9	299
ACOT7	2.1109	8.2	380
CBX3	85.54	42.6	183
U2AF2	5.0259	18.9	475
XRN2	1.9967	2.7	950
DIS3	20.629	13.5	958
RALY	2.1324	17.5	306
SNW1	4.1831	7.4	536
CCT5	7.0795	15	541
TPX2	14.204	13	747
ACIN1	145.79	10.4	1341
KDM2A	5.958	7.7	1162
RAB21	22.895	23.6	225
SNRNP200	11.387	4.9	2136
RBM34	2.3891	6.2	430
CLASP2	1.6079	2	1293
EPB41L3	8.994	7.4	1087
NUP205	4.2315	3.4	2012
TTLL12	4.6218	6.4	644
METAP1	4.1314	8	386
LARP4B	20.609	9.1	738
GANAB	3.2578	3.6	966
FAM120A	2.7163	3.4	1118
FAF2	27.265	25.2	445
DNAJC9	4.8722	15.4	260
ADNP	4.7803	4.6	1102
TARDBP	17.2	25.9	414
CBX5	42.052	54.5	191
RPL13A	5.1704	16.7	203
SRRM2	22.821	6.3	2752
ACOT9	2.0225	7.4	448
MTCH2	10.209	30	303
CIZ1	35.392	22.8	954
RPL36	6.2864	30.5	105
AHCTF1	58.69	13.5	2275
SAMHD1	1.9308	4.2	626
PNKD	1.7214	14.8	385
HIGD1A	5.4901	32.5	107
CHTOP	80.939	32.3	249
SERBP1	235.8	30.5	408
RSL1D1	3.1278	8.1	490
PHGDH	65.261	36.7	533
FBXO2	6.5564	13.8	296
FAM162A	7.333	7.6	154
CKAP2	10.992	7.6	683
PABPC1	79.16	35.2	636
AKAP8L	3.278	7.1	646
SND1	2.2674	3.6	910
RBMX	98.369	45.5	391
PRPF19	14.114	19.2	504
UTP20	1.2694	0.4	2785
ATAD2	36.062	16.4	1390
**UHRF1**	**1.9173**	3.6	**806**
TRA2A	13.749	19.9	282
SEC61A1	2.0643	6	476
DNTTIP2	3.2293	4.3	756
EHD4	5.6122	13.7	541
STOML2	31.968	24.1	356
HP1BP3	4.9031	14.7	553
NSDHL	3.386	9.4	373
HDDC2	4.8913	15.9	204
RPS27L	-2	32	84
NDUFA13	2.8229	20.8	144
MRPS7	1.9919	8.3	242
DDX47	2.8812	9.6	455
TIMMDC1	7.073	16.5	285
**UBR5**	**3.2929**	1.1	**2799**
EIF3L	6.6198	9.6	564
YTHDF2	13.736	7.4	579
RTCB	37.537	28.7	505
HACD3	2.105	8.2	362
TRMT112	3.7074	25.5	125
RAB14	60.379	58.1	215
RAB6B	1.6722	22.4	208
SRRT	2.3314	6	876
NOP58	88.096	31.6	529
C14orf166	5.3574	20.5	244
SF3B6	7.4379	22.4	125
MRPS23	1.7799	13.2	190
ZFR	1.0465	2.6	1074

VPS29	1.6854	4.7	214
RSF1	17.493	7.2	1441
MBD3	2.5958	16.2	291
PELO	13.499	6.5	385
DNAJC10	5.7139	8	793
GAR1	6.9844	16.1	217
RBFOX1	5.7568	8.1	418
TMED9	3.6423	12.8	235
GATAD2A	4.9261	14	633
NSUN2	51.031	15.4	767
TMEM70	2.9233	11.2	260
PHIP	123.44	17.5	1821
MAGOHB	2.8629	13.5	148
ARHGAP17	1.809	3	881
TMEM33	40.921	19.4	247
RIF1	9.173	2.4	2472
ARL8B	4.5097	18.8	186
ATAD3A	46.859	27.5	634
NAT10	3.5237	6.1	1025
WDR33	1.629	1.7	1336
LRRC59	8.5258	19.9	307
NOP10	7.9967	57.8	64
H2AFY2	22.943	39	372
ZNF280C	10.265	13.3	737
LYAR	111.34	29.3	379
VPS35	3.1877	6.3	796
AGK	28.937	37.3	422
DCP1A	2.7284	5	544
ALLC	1.112	2.8	391
CAND1	27.849	15.9	1230
CISD1	1.2138	12	108
DMAP1	1.3644	4	467
ZC3HAV1	10.927	8.6	902
PNO1	2.3117	22.1	252
NCLN	3.0901	4.1	563
HMCES	2.4646	8.6	354
TOMM22	46.914	48.6	142
ADCK3	5.7266	6.6	647
NUP107	2.0814	3.9	925
S100A14	6.1287	25	104
GATAD2B	25.052	20.8	593
NUFIP2	3.4141	8.6	695
CCAR2	4.5494	6.3	923
SRPRB	16.444	24.7	271
RBM25	1.4849	7.4	843
WIZ	2.7429	3.4	794
UBL5	1.3973	24.7	73
AASDHPPT	1.4002	4.9	309
ELAC2	6.0158	9.5	826
RBM15	3.5949	5.7	977
MRPL14	2.4608	9.7	145
PYCRL	4.323	34.3	286
CCDC86	9.1663	8.6	360
WDR77	1.0906	5.4	342
CDC73	6.4767	17.9	531
ZC3H14	1.6935	10	736
TARS2	3.1312	3.9	718
HM13	2.4236	8.4	426
TMX1	2.5092	8.2	280
SFXN3	1.4202	5.1	325
SLIRP	9.2412	53.3	109
EPPK1	1.2102	0.4	5088
GRWD1	1.1525	5.4	446
KATNA1	1.3944	4.3	490
UTP15	1.7062	4.2	518
FAR1	4.767	7	515
FYTTD1	2.0146	9.1	318
POLDIP3	36.556	33.8	438
NOA1	1.9769	2.3	698
NTPCR	17.688	31.6	190
PHF6	14.818	14.2	365
GFM2	7.7599	3.5	779
TUBB6	20.977	31.2	446
GLYR1	7.1179	8.4	553
TUBA1C	71.343	48.9	449
USMG5	13.457	43.1	58
SERPINB12	3.1611	6.2	405
**ZNF598**	**2.3291**	2.8	**904**
MTDH	1.3556	9.8	582
TIMM50	17.224	18.1	456
ADAMTSL1	1.3359	0.5	1762

SFXN1	51.005	30.7	322
H2AFV	13.248	31.2	128
CDCA5	10.156	20.2	252
DCD	3.5203	12.7	121
EXOSC6	2.4708	9.2	272
HIST1H4A	136.75	59.2	103
HIST1H4A	136.75	59.2	103
HIST1H4A	136.75	59.2	103
HIST1H4A	136.75	59.2	103
HIST1H4A	136.75	59.2	103
HIST1H4A	136.75	59.2	103
HIST1H4A	136.75	59.2	103
HIST1H4A	136.75	59.2	103
HIST1H4A	136.75	59.2	103
HIST1H4A	136.75	59.2	103
HIST1H4A	136.75	59.2	103
HIST1H4A	136.75	59.2	103
HIST1H4A	136.75	59.2	103
HIST1H4A	136.75	59.2	103
ZC3H18	3.1196	6	977
EARS2	3.1541	4.9	523
RAVER1	8.4167	4.6	756
HIST2H3A	11.076	37.5	136
HIST2H3A	11.076	37.5	136
HIST2H3A	11.076	37.5	136
NUDCD2	1.4538	15.9	157
DYNLL2	2.1609	12.4	89
ROMO1	7.3248	28.8	79
AMOT	29.373	11.6	1084
HSFY1	1.0908	2.7	NA
PLBD2	10.037	11.4	589
RPL22L1	2.4994	9.9	122
Q7Z7K6	54.748	46.5	272
TUBB	242.21	64.6	444
P51991	79.367	46.8	378
HNRNPUL2	28.829	14.2	747
GAS2L2	4.8671	1.6	880
TTLL10	1.3512	26.7	673
FAM98B	4.7	10.9	433
ZNF326	7.6558	6.5	582
TUBB8	175.97	24.5	444
NDUFS7	7.4456	12.6	213
SBSN	3.2767	15.3	590
KPRP	15.924	15.4	579
TIMM23	29.3	21.5	209
IGLL5	11.74	28.3	214
RBM14	118.39	29.9	669
RBM14	118.39	29.9	339
LUC7L2	3.6309	6.6	113
LUC7L2	3.6309	6.6	392
LUC7L2	3.6309	6.6	458
RPS26	14.03	29.6	115
CBS	4.2005	7.4	551
U2AF1	9.7716	29.2	240
U2AF1	9.7716	29.2	240
CSNK1E	1.669	6.5	416
